# Nanobody: a promising toolkit for molecular imaging and disease therapy

**DOI:** 10.1186/s13550-021-00750-5

**Published:** 2021-01-19

**Authors:** Guangfa Bao, Ming Tang, Jun Zhao, Xiaohua Zhu

**Affiliations:** 1 Department of Nuclear Medicine, Tongji Hospital, Tongji Medical College, Huazhong University of Science and Technology, 1095 Jiefang Ave, Wuhan, 430030 China; 2 Department of Anatomy, School of Basic Medicine, Tongji Medical College, Huazhong University of Science and Technology, 1095 Jiefang Ave, Wuhan, 430030 China

**Keywords:** Nanobody, Molecular imaging, Cancer, Inflammation, Therapy

## Abstract

Nanobodies are the recombinant variable domains of heavy-chain-only antibodies, with many unique properties such as small size, excellent solubility, superior stability, quick clearance from blood, and deep tissue penetration. As a result, nanobodies have become a promising tool for the diagnosis and therapy of diseases. As imaging tracers, nanobodies allow an early acquisition of high-quality images, provide a comprehensive evaluation of the disease, and subsequently enable a personalized precision therapy. As therapeutic agents, nanobodies enable a targeted therapy by lesion-specific delivery of drugs and effector domains, thereby improving the specificity and efficacy of the therapy. Up to date, a wide variety of nanobodies have been developed for a broad range of molecular targets and have played a significant role in patients with a broad spectrum of diseases. In this review, we aim to outline the current state-of-the-art research on the nanobodies for medical applications and then discuss the challenges and strategies for their further clinical translation.

## Background

**Fig. 1:**
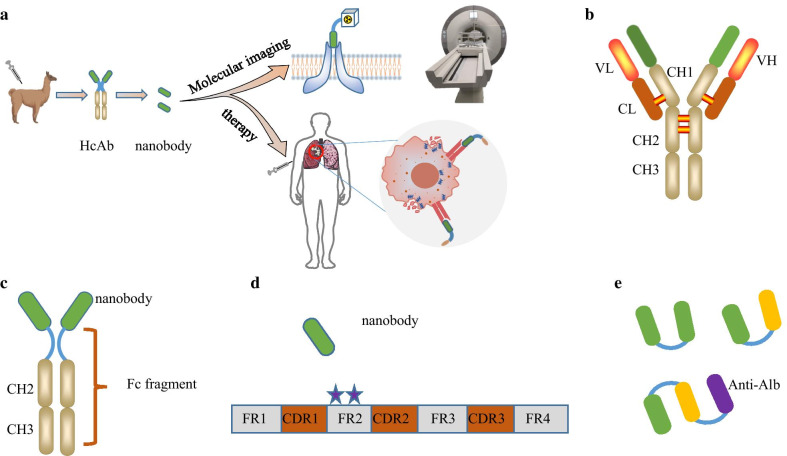
Schematic illustration of mAb, HcAb, nanobody, and multivalent nanobody. (**a**) The application of nanobodies, it has a favorable role for imaging and therapy. (**b**) Classical mAb is composed of two identical light (L) chains and heavy (H) chains. Each heavy or light chain contains two functional domains, i.e., variable region (VR) and one constant region (CR). The difference is that light chain has only one constant region, whereas heavy chain has three or four constant regions. (**c**) HcAb naturally lacks light chains and CH1 domains. Its variable fragment is the nanobody. (**d**) Nanobody consists of four framework regions and three complementarity-determining regions. (**e**) Nanobodies can be produced in a bivalent format, either bivalent-monospecific or bivalent-bispecific. Furthermore, the addition of a third nanobody that binds to serum albumin (anti-Alb) can form multivalent constructs; all these formats can prolong the half-life of nanobodies in the bloodstream

Nanobody (Fig. 1a, d) is the variable domain of heavy-chain-only antibody (HcAbs, Fig. 1a, c) that was first isolated two decades ago from the serum of Camelidae family [1]. The nomenclature of “nanobody” originally adopted by the Belgian company Ablynx® stemmed from its nanometric size, i.e., 4 nm in length, 2.5 nm in width, and only 15 kD in molecular weight [2, 3], which was attributed to the lack of the light chains (L) and heavy chain constant domain (CH) in contrast to the conventional monoclonal antibodies (mAbs, Fig. 1b). The antigen-binding capacity of nanobodies, however, remains similar to that of conventional antibodies for the following reasons. First, the complementarity-determining region 3 (CDR3) of nanobodies is similar or even longer than that of human VH domain (variable domain of heavy immunoglobulin chain). The former consists of 3 to 28 amino acids (AAs), whereas the latter only 8 to 15 AAs. Second, nanobodies can form finger-like structures to recognize cavities or hidden epitopes that are not available to mAbs. This feature not only enhances the binding affinity and specificity of nanobodies, but also enables the discovery of novel pharmacological targets including the receptor-binding pockets or enzymatic active sites [4–6]. Third, nanobodies exhibit excellent stability, hydrophilicity, and water solubility that help maintain their binding affinity across different conditions, which can be further reinforced by mutating key AAs in the framework region (FR2, Fig. 1d) [7–9].

Nanobodies can be quickly excreted via urine in the same way as peptides or small proteins do because their sizes are below the filtration threshold of glomerular membrane of kidney [10–12]. Such a rapid clearance has a two fold impact on nanobody-based imaging. On the one hand, the intensity of background signals drops quickly after the injection of nanobody-derived imaging tracers, which allows early imaging of non-kidney lesions as well as minimizes the "off-target" toxicity [13–15]. On the other hand, the detection of lesions within or next to kidney becomes more challenging. To mitigate the adverse effects on kidney, nanobodies can be modified by glycosylation, PEGylation, or fusion with albumin-binding units to prolong their blood circulation and lower their renal retention [16, 17]. The modification approach also increases the stability and neutralizing capacity of nanobodies. Alternatively, nanobodies can be co-injected with gelofusine, lysine, or monosodium glutamate [18–20], since all these molecules can block nanobodies' binding to megalin, an important transporter for the kidney reabsorption of nanobodies.

Up to date, a wide variety of nanobodies against a broad range of molecular targets have been developed. While showing unparalleled advantages for the noninvasive assessment of molecular targets, the therapeutic efficacy of nanobodies is, however, limited by the lack of Fc fragment. As a result, nanobodies are commonly used as targeting ligands to specifically direct chemotherapy drugs, radionuclides, or toxins toward lesions of interest [8, 21]. In addition, more sophisticated bivalent or bispecific nanobodies (Fig. 1e) have been constructed with higher binding affinity, specificity, and subsequently better therapeutic capacity than their monovalent counterparts [22]. Taken together, nanobodies have proven to be a promising toolkit for diagnosis and therapy of diseases.

## Nanobodies for molecular imaging

Through the labeling with different isotopes or fluorophores, nanobodies can be tracked noninvasively by standard imaging techniques such as single-photon emission computed tomography (SPECT), positron emission tomography (PET), and optical imaging, to provide a sensitive and quantitative visualization of the target-ligand interactions [8, 23, 24]. Noninvasive assessment avoids the trauma of aspiration biopsy and therefore is convenient for repetitive examination or real-time monitoring of disease progression [25]. Several common imaging isotopes (with their abbreviations and half-lives) are given as follows: technetium-99m (^99m^Tc, 6 h), fluorine-18 (^18^F, 110 min), gallium-68 (^68^Ga, 60 min), copper-64 (^64^Cu, 12 h), and zirconium-89 (^89^Zr, 3.3 days) [8, 9]. A unique feature of nanobody-based imaging is that images with high lesion-to-background signal ratios can be obtained at early time points due to the nanobodies' high lesion uptake and rapid blood clearance.

Tumor imaging is by far the most studied area for nanobody-based imaging. The molecular targets for nanobody-based tumor imaging are summarized in Table 1, including epidermal growth factor receptor (EGFR1 or HER1) [26–29], HER2 [23, 30–33], HER3 [34], hepatocyte growth factor (HGF) [35], and carcinoembryonic antigen (CEA) [36]. Most of these targets have been extensively discussed elsewhere [7, 8, 23, 37] and therefore will not be covered in this review. Instead, our focus shifted to the studies that explore the imaging of tumor-associated stroma and including programmed cell death protein-1 (PD-1) and its ligand (PD-L1) [38–40], carbonic anhydrase IX (CAIX) [41], and macrophage mannose receptor (MMR, or CD206) [42–44]. In addition, we also outline the studies about a few inflammatory markers, as exemplified by vascular cell adhesion molecule-1 (VCAM1, or CD106) [45, 46] and V-set immunoglobulin-domain-containing4 (VSIG4) [47].

**Table 1:** Nanobodies for molecular imaging

Target	Nanobody (with the format of nanobody)	Tracer	Maximum uptake of the lesion (with time for imaging post-injection)	References
HER1	8B6 (monovalent)	^99m^Tc	5.2 ± 0.5 %IA/cm^3^ (3 h)	[26]
	7C12 (monovalent)	^99m^Tc	4.55 ± 0.24 %IA/cm^3^ (1 h)	[26, 27]
	7D12 (monovalent, bivalent)	^99m^Tc	4.62 ± 0.36 %IA/cm^3^ (1 h and 3 h)	[26, 27]
	OA-cb6 (monovalent)	^99m^Tc	2.93 ± 0.46 %ID/g (4 h)	[28, 29]
HER2	2Rs15d (monovalent)	^99m^Tc, ^68^Ga, ^18^F	4.23 ± 0.99 %IA/g (1 h)	[23, 32]
	11A4 (with IRDye 800CW)	IRDye 800CW	1.8 ± 0.5 %ID/g (1 h)	[33]
	5F7GGC (monovalent)	^131^I, ^18^F	24.50 ± 9.89 %ID/g (8 h, 1 h)	[84]
HER3	MSB0010853 (monovalent)	^89^Zr	6.2 ± 1.1 %ID/g (24 h)	[34]
HGF	1E2 and 6E10 (fused to albumin)	^89^Zr	8.9% ± 1.0 %ID/g (unknown)	[35]
CEA	NbCEA5 (with humanized nanobody scaffold)	^99m^Tc	7.09 ± 1.36 %IA/cm3 (1 h)	[36]
MMR	α-MMR Nb (monovalent)	^99m^Tc	3.02 ± 0.10 %IA/g (3 h)	[52]
	MMR 3.49 (monovalent)	^99m^Tc, ^68^Ga, ^18^F	2.40 ± 0.46 %IA/g (1 h)	[42–44]
VCAM1	cAbVCAM1-5 (monovalent)	^99m^Tc, ^64^Cu, ^18^F	2.99 ± 0.07 %ID/g (2 h)	[45, 46]
VSIG4	NbV4m119 (monovalent)	^99m^Tc	0.01–0.08 %IA/g (1 h and 3 h)	[64, 65]
PD-L1	Nb109 (monovalent)	^68^Ga	4.94 ± 0.46 %ID/g (1 h)	[69]
CAIX	B9 (with IRDye 800CW)	IRDye 800CW	4.6 ± 0.8 %ID/g (2 h)	[41, 73]

%IA/g: % injected activity per gram tissue, %ID/g: % injected dose of per gram tissue

### Imaging of macrophage mannose receptor

Cancer-related non-resolving inflammation is a hallmark of cancer that leads to the tumor infiltration by different types of immune cells, including tumor-associated macrophages (TAMs) [48, 49]. TAMs reside in hypoxic tumor regions and behave in a context-dependent manner. On the one hand, TAMs can present tumor-associated antigens to stimulate anti-tumor immune responses and enhance the function of cytotoxic T lymphocytes. On the other hand, the excessively activated TAMs can promote tumor proliferation and progression. Accordingly, TAMs are often categorized into the canonically activated anti-tumor M1 phenotype and the alternatively activated pro-tumor M2 phenotype [50, 51]. It is noteworthy, however, that TAMs are a group of heterogeneous cells showing phenotype plasticity that can adapt to surrounding microenvironment, while the M1/M2 dichotomy is just a simplified stratification of TAM subsets [50]. TAM subsets can be noninvasively distinguished by their surface markers specific to their surrounding microenvironment. Macrophage mannose receptor (MMR) is one of such markers [52].

Movahedi K et al. isolated anti-MMR nanobody clone 1 (Nb cl1, also known as α-MMR nanobody) from an immune nanobody phage-display library. Biodistribution studies in wild-type mice verified the uptake of α-MMR nanobody in organs or tissues where macrophages commonly reside, including cardiac muscle, bone marrow, spleen, liver, etc. In contrast, only background levels of tracer uptake were detected in MMR-deficient mice. The tumor targeting was further improved by using the bivalent construct of Nb c11, which not only increased its binding affinity but also prolonged its blood circulation [52]. In addition, co-injection of its unlabeled bivalent construct could further reduce its uptake in non-tumor organs without affecting the uptake in tumors. Of note is that this method could be extended to other types of nanobodies and greatly enhanced the translational potential of nanobody-based imaging.

Another anti-MMR nanobody—anti-MMR 3.49—was identified from 27 clonally unrelated nanobodies after repeated selections for high tumor accumulation and low liver or spleen uptakes. Interestingly, the biodistribution of anti-MMR 3.49 was similar to that of Nb cl1, with high uptake in MMR-expressing organs and tissues and negligible accumulation in MMR-deficient mice. Moreover, the radioisotopes can also make a great impact on the biodistribution pattern of nanobodies: the ^18^F-labeled ones had 20-fold lower renal uptake than their ^99m^Tc-labeled counterparts at 3 h after injection. The pattern difference was attributed to their distinctly different behaviors in vivo in terms of activity, charge, and metabolism. For example, the renal metabolite of ^18^F-labeled nanobody was hydrophobic and therefore could diffuse out of the tubular cells and be readily cleared from the body [42–44]. Nevertheless, the preclinical data highlight the potential of anti-MMR 3.49 for tumor staging and prognosis prediction [42]. Several recent clinical trials are evaluating the efficacy of colony-stimulating factor-1 (CSF-1), as well as the inhibitors and antibodies of CSF-1 receptors, in modulating TAM. Therefore, techniques for the noninvasive characterization of TAM are expected to be useful and clinically important should these therapeutic strategies show any marked effects [53–55].

In addition to tumor imaging, anti-MMR nanobodies are also useful for evaluating inflammatory diseases, e.g., atherosclerosis and rheumatoid arthritis, where macrophage polarization is commonly observed [43, 56]. Varasteh et al. evaluated ^99m^Tc-labeled anti-MMR3.49 for the in vivo imaging of atherosclerosis models. Compared to isotype control nanobody, the ^99m^Tc-labeled anti-MMR 3.49 showed significantly higher uptake in all the aortic segments of ApoE-negative mice. The difference of tissue uptake was diminished in MMR-knockout mice or in case of competition studies when unlabeled anti-MMR3.49 was injected before the labeled ones. Immunofluorescence staining further confirmed that the MMR^+^ macrophages mainly located in the adventitial layer adjacent to intimal lesions, the fibrous cap layer, and shoulder region of the plaques [43, 44]. Senders ML et al. developed an integrated protocol of using PET and magnetic resonance imaging (MRI) to noninvasively evaluate the distribution of MMR^+^ macrophages during the evolution of atherosclerosis. After injecting ^68^Ga-labeled MMR3.49, the intensity of PET signals from the aorta areas gradually increased as the disease progressed, indicating the recruitment of MMR^+^ macrophages. T2-weighted MRI and dynamic contrast-enhanced (DCE) MRI showed a concurrent swelling of vessel wall and an increase in vessel permeability. There was a significant correlation between the area of vessel wall and the uptake values of ^68^Ga-MMR3.49 (*r* = 0.55, *p* = 0.0002), confirming the accumulation of MMR^+^ macrophages during the progression of atherosclerosis. On account of its robust and noninvasive readouts, this dual-imaging protocol is an attractive approach to plaque imaging and quantification of atherosclerosis hallmarks [44, 57].

### Imaging of the vascular cell adhesion molecule-1

The inflammatory process leading to the development of vulnerable atherosclerotic lesions is often accompanied by leukocyte infiltration, during which the leukocytes extravasate across the arterial wall after a process of rolling, adhesion, and transmigration, and then cluster at the inflammation site [58]. A pivotal regulator of this process is the vascular cell adhesion molecule-1 (VCAM1, also known as CD106), a receptor for the very late antigen-4 (VLA4) at leukocyte surface. Therefore, VCAM1 has attracted much attention as the target for a noninvasive detection of inflammation [45].

Broisat et al. prepared 10 anti-VCAM1 nanobodies (coded cAbVCAM1-1 to cAbVCAM1-10). The cAbVCAM1-5 formulation, which exhibited the highest uptake at inflammation site as well as the highest lesion-to-normal tissue ratio, was then selected as the probe for subsequent single-photon emission computed tomography/computed tomography (SPECT/CT) imaging. ^99m^Tc-cAbVCAM1-5 accumulated at VCAM1^+^ atherosclerosis lesion, while its non-targeting counterparts only showed baseline signals. More importantly, there was a significant correlation between the uptake of ^99m^Tc-cAbVCAM1-5 and the relative volume of atherosclerosis lesion [45, 46]. In other two studies, PET/CT (PET/computed tomography) imaging further established that radioactivity accumulation at the lesion site could predict the development of advanced-stage atherosclerosis [57, 59]. On the other hand, PET/MR imaging using [^64^Cu]-cAbVCAM1-5 showed higher tracer uptake at early-stage atherosclerosis lesions than those at advanced stage [57]. Such discrepancy may arise from the fact that leukocyte infiltration happens in both early-stage atherosclerosis and advanced vulnerable atherosclerotic lesions. In spite of the ambiguity, a phase I clinical trial has been launched to evaluate cAbVCAM1-5 for the assessment of atherosclerosis. Intriguingly, among the various radio-labeled nanobody ^99m^Tc-labeled cAbVCAM1-5 showed the highest lesion uptake, followed by the ^68^Ga- and ^18^F-labeled tracers, demonstrating that radioisotope did have a significant impact on the biodistribution of nanobodies [46]. Meanwhile, plaques detectability was improved by using restrained complexing agents (RESCA) as the radioisotope chelators, which allowed faster ^18^F-labelling and yielded significantly higher plaque-to-brain and plaque-to-heart ratios [60]. VCAM-1 is a good target for the detection of existing atherosclerosis due to its highest abundance among atherosclerosis-related adhesion molecules. It is also useful for the detection of activated endothelium at the risk of developing plaques, because VCAM-1 is a major participant during the initiation of atherosclerosis [61, 62]. Simultaneously, given that VCAM1 is involved in many other immunological disorders, the horizon of anti-VCAM1 nanobody applications can be further expanded [63].

### Imaging of V-set immunoglobulin-domain-containing4

V-set immunoglobulin-domain-containing4 (VSIG4) is a membrane protein that belongs to the complement receptor of the immunoglobulin superfamily (CRIg). Unlike MMR that can be detected in liver sinusoidal endothelial cells, VSIG4 expression is confined to the surface of a subset of resident macrophages including that of liver Kupffer cells [64]. The VISG4 expression on macrophages is substantially upregulated during inflammation; therefore, it is considered a more specific biomarker than MMR for inflammatory lesions.

^99m^Tc-NbV4m119 is an optimized nanobody-based tracer that specifically targets CRIg, including VISG4. Its accumulation at inflammatory lesions correlated significantly with the clinical score. Moreover, ^99m^Tc-NbV4m119 was detected in the asymptomatic joints of collagen-induced arthritis (CIA) mice as early as 9 days before the inflammation onset. Taken together, these results demonstrate that ^99m^Tc-NbV4m119 is a promising tool to predict the occurrence and grade of CIA and thereby allows early treatment for the disease [47]. In addition, ^99m^Tc-NbV4m119 can also noninvasively visualize the change of Kupffer cells during inflammation, because its target VISG4 is specifically present in Kupffer cells. Indeed, the liver uptake of NbV4m119 was significantly reduced after the depletion of all phagocytes by injecting clodronate liposomes, while the liver uptake of anti-MMR nanobodies remained the same. This difference can be attributed to the fact that MMR is abundantly expressed on different types of liver cells, whereas VIG4 is confined to Kupffer cells [64]. In a concanavalin A (ConA)-induced acute liver injury model, it was reported that the ^99m^Tc-NbV4m119 signals at liver region reached nadir at 24 h after ConA challenge and then slowly recovered at 48 h. The imaging findings corroborated well with the changes in the liver expression of VISG4 and the number of Kupffer cells [64, 65]. In another study on non-alcoholic steatohepatitis (NASH), it was also confirmed that ^99m^Tc-NbV4m119 could track the dynamic changes of Kupffer cells in a noninvasive manner and thereby closely observe the occurrence, development, and regression of liver inflammation [65]. In summary, we conclude that VSIG4 is a more-specific biomarker for hepatic inflammatory disorders, and further clinical translational studies are warranted.

### Imaging of programmed cell death ligand-1

Programmed cell death protein-1 (PD-1) and its ligand (PD-L1) belong to the family of immune checkpoint molecules that can prevent immune overstimulation and maintain self-tolerance [38, 66]. However, tumor cells may also overexpress PD-L1 to suppress the activity of effector T cells and thereby resist immunotherapy [67]. Since the blockade of the PD-1/PD-L1 axis has shown promising efficacy in cancer treatment, there is a growing need to stratify patients or predict prognosis by the noninvasiveness assessment of PD-1 or PD-L1 expression. Several nanobody-based tracers are being investigated in this regard.

Zhang F et al. produced a heavy-chain-only antibody (KN035) that specifically targets human PD-L1. In a competitive binding assay, KN035 displaced human PD-1 more effectively than durvalumab, a commercial anti-PD-L1 monoclonal antibody. There was neither any cross-reaction with human PD-L2 nor mouse PD-L1 [38]. Based on these ligand-binding results, Li D et al. went on to prepare ^89^Zr-labeled KN035 for PET imaging of a human glioma xenograft model in mouse. The blood activity peaked at 1 hour post-injection and decayed rapidly thereafter. The tumor-to-muscle contrast ratio reached 5.64 ± 0.65 at 24 h post-injection and then slowly plateaued to 7.70 ± 1.37 at 120 h. Therefore, the ^89^Zr-labeled KN035 allows PET imaging of tumor at as fast as 24 h after tracer injection, as compared to the 3 to 5 days of waiting time needed for radiolabeled whole monoclonal antibodies [39]. Another study from the same group further confirmed that KN035 was a specific and sensitive probe to assess PD-L1 level, as evidenced by the different tracer accumulation between the control and EGFR-tyrosine kinase inhibitor (TKI)-treated groups [40]. Moreover, KN035 has shown promising anti-tumor efficacy in a phase I study in the USA, indicating that it can be used in combination with therapeutic nuclides [68].

Lv G et al. developed another PD-L1-targeting nanobody (Nb109), with a blood half-life of only 49.79 min. Its tumor uptake reached 5.32 ± 0.47 %ID/g at only 10 min post-injection. The tumor-to-muscle ratio peaked at 11.03 ± 0.36 at 1 hour post-injection and then slowly decreased to 6.76 ± 0.41 at 2 h post-injection. Interestingly, the bind curve of Nb109 to PD-L1 was not affected by adding either PD-1 or KN035. The uptake of Nb109 in PD-L1^+^ cells did not diminish even in the presence of 1000-fold excess of KN035 [69]. These observations indicate that Nb109 has a different PD-L1-binding epitope from those of PD-1 or KN035. As a result, it is probable to further improve the binding affinity and specificity by constructing a bivalent molecule linking Nb109 and the antigen-binding domain of KN035. Taken together, both KN035 and Nb109 are potential candidates to stratify patients before treatment with FDA-approved PD-1/PD-L1 inhibitors such as nivolumab, pembrolizumab, atezolizumab, and durvalumab.

### Imaging of carbonic anhydrase IX

Hypoxia in the tumor microenvironment, caused by the abnormal intratumoral blood vessels, can promote tumor growth through activation of the hypoxia-inducible factor 1 and 2 (HIF-1/2) signaling [70, 71]. Carbonic anhydrase IX (CAIX) belongs to one of the most upregulated targets downstream of HIF-1/2 signaling and plays a pivotal role in posing an acidic microenvironment to promote cancer progression and metastasis [71, 72]. In addition, CAIX is ubiquitously expressed in hypoxic tumors regardless of tumor types, making it a feasible target for imaging and therapy [41].

Van Brussel et al. prepared an optical imaging tracer based on an anti-CAIX nanobody B9. Its binding specificity was verified by a CAIX expression-dependent uptake of nanobody B9 in CAIX-expressing cells, as well as a blocking study using human recombinant CAIX ectodomain. The IRDye800CW-labeled B9 (B9-IR) was then evaluated in a mouse orthotopical xenograft model bearing CAIX-overexpressing ductal carcinoma in situ cells. The tumor-to-normal tissue ratio reached 4.3 ± 0.6 at 1 hour post-injection and remained similar for the next 8 h. Therefore, B9-IR holds great promise for the fluorescence-guided tumor resection, since the surgery can be performed on the same day of tracer injection [41].

B9-IR also can be co-injected with another nanobody that targets a different tumor marker and is labeled with a non-overlapping fluorescence dye, e.g., co-injecting B9-IR with fluorescence-labeled anti-HER2 nanobody 11A4. This approach not only further increased the tumor-to-normal tissue ratio but also allowed a simultaneous visualization of two tumor markers and therefore substantially improved the sensitivity and specificity of locating tumor metastasis [73].

Of note, as a potential pan-cancer target, CAIX holds great promise for the visualization of local hypoxia and acidosis in tumor microenvironment, and so for the dampening of tumors while conjugating with therapeutic radionuclides.

## Nanobodies for therapy

Nanobodies, as the smallest naturally derived antigen-binding fragments, share both similarities and differences with their parent monoclonal antibodies when used as therapeutic agents. Similar to monoclonal antibodies, nanobodies can bind to transmembrane receptors or soluble ligands to regulate downstream signaling pathways [74]. The long CDR domain of nanobodies can bind to epitopes that are not accessible to monoclonal antibodies and thereby facilitate the discovery of novel pharmaceutic targets [4–6]. By manipulation of their encoding genes, multivalent or multi-specific nanobodies can be prepared to exert similar or stronger binding than conventional antibodies. However, the lack of Fc domains deprives nanobodies of immune cell-mediated or complement-dependent cytotoxicity. As a result, they are considered more suitable as targeting moieties for delivering therapeutic drugs, radionuclides, toxins, and peptides [8, 22]. Besides, nanobodies can also be used for tumor vaccination strategies and CAR-T cell therapy [75]. Vaccination based on nanobodies can be delivered directly to antigen-presenting cells (APC), and nanobody chimeric receptor can target to and induce the lysis of tumor-associated antigen-positive cells [76]. Recent studies have demonstrated the potential of nanobodies in the treatment of diseases; the recently approved nanobody caplacizumab was a belated but potentially lasting landmark event for nanobodies [77, 78], and an increasing number of clinical trials about nanobodies is ongoing [78].

### Nanobody-targeted radionuclides

**Fig. 2:**
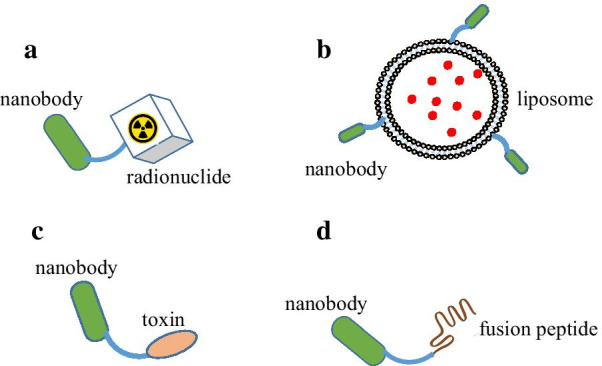
Nanobody-based targeted disease therapy. Owing to their superior target specificity and binding affinity, nanobodies often serves as targeting moieties and brings effector domains or drugs to lesions of interest, including radionuclide (**a**), drugs (**b**), toxins(**c**), and fusion peptides (**d**)

Radiation therapy, including external beam radiation and targeted radionuclide therapy, is one of the three pillars for cancer therapy [79]. External beam radiation cannot treat disseminated lesion and often cause lateral damage to normal organs. In contrast, targeted radionuclide therapy can selectively deliver radiation dose to cancer cells using radiopharmaceuticals consisted of a targeting ligand (e.g., monoclonal antibody) and a therapeutic radionuclide (Fig. 2a) [80, 81]. Currently, three types of radionuclides are in clinical use or preclinical evaluation: β^−^ particles, Auger electrons, and α particles, which damage DNA either by direct ionization of DNA strands or through the generation of reactive oxygen species (ROS) [82]. Nanobodies, on the other hand, are excellent alternatives to monoclonal antibodies as the targeting ligand due to their superior tissue penetration, binding affinity, and specificity [83]. Several relevant studies are discussed as follows.

2Rs15d is a HER2-specific nanobody that binds to a different domain in comparison with commercial therapeutic monoclonal antibodies including trastuzumab and pertuzumab [84]. Therefore, 2Rs15d can be used simultaneously with the two antibodies without affecting their target binding. Pruszynski M et al. labeled nanobody 2Rs15d with an α-emitting radionuclide actinium-225 via the chelator 2-(4-isothiocyanatobenzyl)-1,4,7,10-tetraazacyclododecane-1,4,7,10-tetraacetic acid (p-SCN-Bn-DOTA). The resultant ^225^Ac-DOTA-2Rs15d exhibited 60 to 70 times higher uptake in HER2^+^ cells than in HER2^−^ cells. Although ^225^Ac-DOTA-2Rs15d had lower tumor uptake than its monoclonal antibody counterparts, its faster accumulation in tumor is more favorable for the targeted delivery of radiation dose [84, 85]. In another study, [^131^I]-2Rs15d significantly prolonged mouse survival in a HER2-positive tumor xenograft model (137.5 days vs. 93.5 days, *P* < 0.05). Adding trastuzumab to [^131^I]-2Rs15d further increased the median survival by another 30 days. Importantly, the accumulated radiation dose at kidney was substantially reduced after adding a residualizing prosthetic linker, N-succinimidyl 4-guanidinomethyl-3-[*I] benzoate (SGMIB), between 2Rs15d and ^131^I. As a result, the injection dose can be further increased to improve the therapeutic efficacy as long as the radiation dose at kidneys is below the renal toxicity threshold. Encouraging results were recently been achieved in another study on the application of [^177^Lu]-labeled 2Rs15d, where 5 out of 8 mice-bearing minimal residual or micro-metastatic tumors experienced complete tumor regression after treatment with [^177^Lu]-labeled 2Rs15d [19].

5F7GGC is another anti-HER2 nanobody that is internalized more quickly than 2Rs15d after binding to the HER2 receptor. The tumor uptake of 5F7GGC peaked at 24.50 ± 9.89 %ID/g at 2 h after injection, much higher than that of 2Rs15d. Conjugation of the SGMIB linker can further accelerate its blood clearance, leading to a tumor-to-normal organ ratio above 50 and a tumor-to-kidney ratio of 2.0 ± 0.5 [86]. Because the internalized nanobody generally has higher uptake and longer cellular retention, theoretically, 5F7GGC might be the superior candidate for targeted radionuclide therapy though research about it is lagging in comparison with the development of 2Rs15d.

### Nanobody-mediated drug delivery system

It is well established that specific delivery of toxins and chemotherapy drugs to tumors could not only improve therapeutic efficacy but also decrease side effects. A typical platform of targeted drug delivery is  composed of targeting ligand, drug carrier, and pharmaceutical drug [87]. Nanobodies exhibit several advantages compared to the monoclonal antibody-derived targeting ligands. First, nanobody-conjugated drug carriers (Fig. 2b) are cleared more quickly from blood and therefore are less toxic to the "off-target" normal organs [13–15]. Second, nanobody-conjugated drug carriers are less immunogenic because they lack Fc domains that can active immune cells or the complement system. Lastly, once binding to their receptors, the multiple nanobodies on the same carrier could induce the dimerization and subsequent internalization of the receptors and therefore increase the cellular uptake of the drug payloads. Polymers, liposomes, micelles, and albumin are several categories of frequently used drug carriers [88]. Besides the conventional chemotherapeutic drugs (e.g., doxorubicin), pathway inhibitors and photosensitizers also can be incorporated in drug carriers in a targeted delivery platform [88–90]. Several of these examples are discussed below.

Oliveira et al. conjugated anti-EGFR nanobody EGa1 to PEGylated liposome and studied the resultant formulation, EGa1-L, in terms of EGFR downregulation [91]. EGa1-L bound to several monomeric EGFR simultaneously, which was followed by EGFR dimerization and subsequent internalization via the endosome route. Due to the stability of EGa1-L at acidic pH, the complex of EGa1-L and EGFR remained intact in the endosomes and therefore facilitated EGFR degradation as the endosome matured. In contrast, engineered fragments of monoclonal antibodies, e.g., scFV, are prone to acidification-induced disassembly. The complex of scFV-conjugated liposome and EGFR was therefore more likely to dissociate in endosomes and less effective in degrading EGFR. On the other hand, the EGa1-L liposome formulation was more effective than the free EGa1 nanobody in EGFR degradation. This observation once more underscores that the dimerization and internalization of EGFR induced by multiple EGa1 nanobodies on the same liposome are the key steps for downregulating EGFR. In the follow-up study, the same group conjugated AG538, an anti-insulin-like growth factor 1 receptor (anti-IGF1R) inhibitor, and EGa1 on the same liposome [92]. The resultant EGa1-AG538-L simultaneously inhibited EGFR and IGF1R and was more potent in the inhibition of cancer cell proliferation than the physical mixture of EGa1 and AG538. The blockade of the cross talk between EGFR and IFR1R signaling by EGa1-AG538-L may also alleviates the tumor resistance when EGa1 or AG358 is used alone.

Heukers R conjugated a traceable photosensitizer (IRDye700DX) to two anti-EGFR nanobodies, 7D12 and 7D12-9G8, without affecting their binding properties. The resultant conjugates were selectively taken up by EGFR-expressing cells and ablated the cells effectively upon illumination with near-infrared lasers. 7D12-9G8-IRDye700DX exhibited higher phototoxicity in vitro (half maximum inhibitory concentration (IC50) 0.6 ± 0.06 nM vs. 2.3 ± 0.7 nM). In contrast, 7D12-IRDye700DX exhibited better anti-tumor efficacy in vivo. This discrepancy can be attributed to the different biodistribution profiles of the two conjugates. 7D12-IRDye700DX was smaller and therefore had a better tumor penetration and a more homogeneous distribution within tumor [93]. Therefore, the therapeutic efficacy of nanobody-photosensitizer conjugates is determined not only by the overall amount of injected dose, but also their intratumoral distribution. In addition to direct tumor killing, the phototherapy with 7D12-IRDye700DX also induced tumor infiltration by immune cells. Taken together, the nanobody-derived delivery system enables the precision therapy by photosensitizers with higher curative potential.

### Nanobody-based immunotoxin

Toxin, including plant-derived ones (e.g., ricin, abrin, and gelonin) and bacterial protein-derived ones (e.g., pseudomonas exotoxin and diphtheria toxin), can effectively kill tumor cells regardless of their cell-cycle phases and therefore are promising tools to treat chemoresistant tumors [94–98]. Targeting moieties such as monoclonal antibodies have been conjugated to the toxins, and the resultant product, termed as immunotoxins, can selectively accumulate in tumor regions with minimal side effects [99]. Several monoclonal body-based immunotoxins, including mylotarg, have been approved for treating cancer patients. However, the immunogenicity of monoclonal antibody-based immunotoxins prevented their continual dosing. The large size of monoclonal antibodies also hinders the tumor penetration of corresponding immunotoxins. Therefore, nanobodies appear to provide a viable alternative as the targeting moiety for immunotoxins (Fig. 2c).

CD7 is a cell surface glycoprotein of the immunoglobulin superfamily. It is overexpressed on hematologic cancer cells and rapidly internalized after binding to immunotoxins [100]. Tang et al. constructed immunotoxins based on monovalent and bivalent anti-CD7 nanobodies and coded them as PG001 and PG002, respectively [101]. Both PG001 and PG002 induced specific apoptosis of CD7^+^ leukemia cell lines (Jurkat and CEM), while the latter showed higher cell-binding affinity, longer half-life, and higher therapeutic efficacy. The median survival of PG002-treated animals was 10 days longer than that of PG001. However, their anti-tumor potential is still hindered by immunogenicity and lysosomal degradation of toxins. To overcome these problems, humanized PG002 (also known as dhuVHH6-PE38) was prepared by linking the CDR of VHH6 to a commonly used humanize nanobody scaffold (h-NbBcII10FGLA). Animals treated with dhuVHH6-PE38 exhibited no significant loss of body weight other adverse symptoms. Another variant, dVHH6-PE-LR, was constructed, in which the lysosome-sensitive sites of toxins were deleted, only to show worse anti-tumor efficacy. Taken together, dhuVHH6-PE38 is now the leading candidate in the clinical translation for leukemia therapy [102].

### Nanobody-peptide fusions

The lack of Fc domain is a major limit to the therapeutic efficacy of nanobodies. To overcome this limit, nanobodies can be conjugated with another protein or peptide to form multifunctional protein/peptides (Fig. 2d). Site-specific conjugation via a C-terminal cysteine has been developed to control the reaction sites of the two effector domains and therefore minimize the interference with the nanobodies' binding properties. The resultant conjugates are able to specifically accumulate in their targets under the guidance of nanobodies and exert their functions.

Fc fragments are the most frequently conjugated effector domains. Bobkov et al. fused the dimer of three anti-CXCR4 nanobodies (VUN400, 401, and 402, respectively) with the second and third constant domains (CH2-CH3) of a human IgG1 heavy chain [103]. The resultant conjugates were more potent in displacing CXCL12, the natural ligand for CXCR4, while VUN402 as a monovalent nanobody failed to displace CXCL12. In addition to an increased inhibition of the CXCL12-mediated signaling, the nanobody-Fc conjugates also exhibited Fc-mediated toxicities similar to antibody-dependent cytotoxicity (ADCC). VUN400-Fc bound to effector  cells via Fc receptors (e.g. FcγRIII or CD16) and subsequently induced cell degranulation. Activation of the complement system was also observed. In an in vitro complement-dependent cell death (CDC) assay, only VUN400-Fc but VUN400 nor another irrelevant nanobody-Fc construct induced CDC-mediated death of CXCR4^high^ cells. Taken together, incorporation of Fc domain proves to be a feasible strategy to enhance the therapeutic efficacy of nanobodies.

Nanobodies with fusion domains that induce cluster and/or proliferation of effector cells, e.g., natural killer (NK) cells or cytotoxic T lymphocytes (CTLs), can recruit these immune cells into tumor microenvironment to kill cancer cells. Such fusion domains often target the surface markers of immune cells such as CD16 for NK cells and CD3 for T cells [104]. It should be noted, however, the presence of effector cells is a prerequisite for this strategy to function. Li et al. constructed a bispecific nanobody by linking an anti-CEA and an anti-CD16 nanobody and conjugated each with a mutated human IgG1 Fc fragment [104]. The construct recruited NK cells to tumor lesions to exert significantly higher cytotoxicity to cancer cells than the monovalent anti-CEA-Fc or anti-CD16-Fc. The anti-tumor efficacy is NK-dependent: complete tumor regression was observed in the presence of NK cells, while depletion of NK cells abolished the anti-tumor efficacy. Similarly, the anti-CEA/CD3-Fc bispecific nanobody potently recruited CD3^high^ T cells [17]. Similarly, another fusion protein-anti-CEA-IL15 (interleukin-15) also exhibited much more potent in vivo than the monovalent anti-CEA-Fc, due to the recruitment of CD8^+^ T cells derived by IL15. In a colon cancer mouse model, anti-CEA-IL15 at the dose of 1 µg/mouse inhibited tumor growth by more than 80% without causing significant weight loss or other apparent toxicities [105]. The recruitment and enrichment of effector cells theoretically is a favorable match with immune checkpoint blockade (ICB), because ICB-based therapy requires the presence of effector cells in the tumor microenvironment [106]. Hidde L Ploegh et al.combined a PD-L1-blocking nanobody with chemokine CCL21. This fusion construct not only targeted PD-L1-expressing tumor cells but also facilitated dendritic cells to transmigrate through lymphatic endothelium and home toward these tumor cells [106]. Intriguingly, this approach can extend to other chemokines and nanobodies and are expected to improve therapeutic effects.

## Conclusions and perspectives

In this manuscript, we have reviewed the state-of-the-art technologies using nanobodies for diagnosis and therapy. With the current trend to integrate diagnosis and treatment, nanobodies seem to have a favorable role for this new era: in tumor diagnosis, in assessment and prediction before tailoring and staring a therapeutic protocol, in dynamic monitoring during treatment, in the detection of possible niche for reoccurrence of tumor [8]. Along this line, nanobodies may also be useful for monitoring various other diseases such as amyloidosis, viral infections, far more than what we mentioned in this review. Apart from traditional PET/CT or SPECT imaging, the application of nanobodies can be further extended to super-resolution imaging to study protein structure, functions, and protein-protein interaction [107]. By applying anti-FP (fluorescent proteins) to deliver bright organic fluorophores to FP-tagged protein, images of subcellular structure including nuclear pore complex, tubulin, and vimentin could be gotten, with nanometer spatial resolution and minimal linkage error yet without interfering with the native organization of these proteins. Intriguingly, virtually any known protein can be visualized through this scheme [108–110]. Additionally, as they provide access to conformational epitopes in concave and hinge regions, nanobodies have been used to freeze dynamic proteins into single functional conformations. Thus, the dynamic changes in the structures and functions of intracellular proteins can therefore be well studied.

The advent of “radiopharmaceuticals” further making the combined applications of imaging and therapy become feasible; the conjugated nuclide can be traced by PET or SPECT machine and can emit short-range radiation for therapeutic purposes simultaneously. Moreover, via modifications and functionalization (e.g., PEGylation and conjugation to the Fc domain, peptides, drugs, and toxins), we can take advantage of nanobodies to function as a targeting moieties and, meanwhile, to overcome the therapeutic limitations brought by the lack of Fc domain [111]. Additionally, nanobody-based fluorescence-guided cancer surgery provides the surgeon with real-time visualization, precise and specific identification of tumors and then helps them find (micro) metastases as well as occult tumor cells in the intraoperative context, even those submillimeter islands of tumor cells [33, 41]. There is evidence that lesions missed by eyes can be clearly visualized while using near-infrared (NIR) fluorescence-guided surgery imaging system (like Artemis). Therefore, surgeon can resect tumors more thoroughly, improving the prognosis of patients to a great extent. Very recently, it has been also shown that nanobodies play a favorable role in tumor vaccination strategies and chimeric antigen receptor T cell (CAR-T) therapy [76]. Via constructing nanobody-expressing lentiviral vectors (LVs), tumor-associated antigen could be delivered to APCs in an Nb-dependent and APC-specific manner [112]. In another approach, CAR-T cells, which are engineered to consist of nanobodies as the targeting domain, are effective in eliminating tumors [113, 114]. Likewise, this approach can also be extended to CAR-NK cells, a focus of current tumor treatment.

At present, the biggest hurdle for clinical translation of nanobodies is their high uptake in kidneys. The kidney accumulation not only lowers the sensitivity in detection of lesion close to kidney, but may also cause nephrotoxicity [23, 52]. It should be noted, however, that, kidney uptake is caused by the combined effects of the intrinsic characteristics of nanobodies, the chemical characteristics of chelators and radionuclides, and the stability of radiolabeled compound. Strategies to reduce renal retention are summarized in Table 2. These methods will greatly facilitate the clinical translation of nanobodies. Taken together, nanobodies are a versatile toolkit that can play a central role in clinical applications and basic science.

**Table 2:** Overview on the nanobody-based applications, their advantages and drawbacks, as well as solutions at present

Function and target	Combined with	Advantages	Disadvantages	Solution	References
Antagonist: EGFR, CXCR4, P2X7, HGF	-	(i) Small size (ii) High solubility and stability (iii) Excellent tissue penetration (iv) Recognizing new targets (v) Using together with mAbs	(i) Fast blood clearance (ii) High renal uptake (iii) Lack of Fc (iv) Immunogenicity	(i) Constructing multivalent nanobody (ii) Co-injecting with cationic amino acids (iii) Conjugating with an anti-albumin unit (iv) Linking with effector domains	[35, 41]
Nanobody-based radionuclide: HER2	^225^Ac,^131^I, ^177^Lu	(i) Fast blood clearance (ii) Suited for conjugation	(i) High renal uptake (ii) Radiation toxicity to healthy cells	(i) Constructing multivalent nanobody, co-injecting with cationic amino acids, conjugating with an anti-albumin unit (ii) Linking with residualizing prosthetic group such as SGMIB (iii) Selecting high-affinity and high-internalization nanobody	[82, 83]
Nanobody-mediated drug delivery system: EGFR, HER2	(i) Pharmeceutic carriers (ii) Chemotherapeutic drugs	(i) Can act as antagonist itself (ii) High specificity (iii) Suited for conjugation	(i) Fast blood clearance (ii) The drug can damage normal cells	(i) Encapsulation in carriers, (ii) PEGylation	[86, 87]
Nanobody-based immunotoxin: EGFR, CD7	(i) Plant toxins (ii) Bacterial protein toxins such as PE and DT	(i) Lethal to cells in all phases (ii) High efficacy	(i) Immunogenicity (ii) Lysosome-sensitive sites in toxin part	(i) Linking nanobody with humanized nanobody scaffold (ii) Deleting lysosome-sensitive sites	[98–100]
Nanobody-peptide fusions: EGFR, DR, CEA	(i) The ligand of death receptor (TRAIL) (ii) Fc domains (iii) Cytokine	(i) Inducing ADCC and CDC (ii) Specifically recruiting effector cells to lesions	Fast blood clearance	(i) Constructing multivalent or nanobody, (ii) Glycosylation modification (iii) Crucial amino acid mutation in FR2	[17, 102–104]

## Search strategy and selection criteria

Data for this review were identified by searches of NCBI, PubMed, and references from relevant articles using the search terms “cancer,” “tumor,” or “inflammation” and “nanobody,” “VHH,” or “single-domain antibody” in the abstract, title, or keywords. We choose the literature which is published after 2010, occasionally with ones before.

## Data Availability

The data cohorts used in this review are available from the corresponding author on reasonable request.

## Abbreviations

HcAbs: Heavy-chain-only antibodies
mAbs: Monoclonal antibodies
Fc: Crystalline fragment
Nb: Nanobody
AAs: Amino acids
FR: Framework region
CDR: Complementarity-determining region
CH: The heavy chain constant domain
VH: Variable domain of heavy immunoglobulin chain
SPECT: Single-photon emission computed tomography
PET: Positron emission tomography
^99m^Tc: Technetium-99m
^18^F: Fluorine-18
^68^Ga: Gallium-68
^64^Cu: Copper-64
^89^Zr: Zirconium-89
^177^Lu: Lutetium-177
^131^I: Iodine-131
^125^I: Iodine-125
EGFR: Epidermal growth factor receptor
HGF: Hepatocyte growth factor
CEA: Carcinoembryonic antigen
PD-1: Programmed cell death protein-1
CAIX: Carbonic anhydrase IX
MMR: Macrophage mannose receptor
TAM: Tumor-associated macrophages
VSIG4: V-set immunoglobulin-domain-containing 4
CRIg: Complement receptor of the immunoglobulin superfamily
MRI: Magnetic resonance imaging
DCE: Dynamic contrast-enhanced
KCs: Liver Kupffer cells
ConA: Concanavalin A
NASH: Non-alcoholic steatohepatitis
VCAM1: Vascular cell adhesion molecule-1
PD-L1: Programmed cell death ligand1
HIF-1/2: Hypoxia-inducible factor 1 and 2
CIA: Collagen-induced arthritis
SGMIB: N-succinimidyl 4-guanidinomethyl-3-[*I]benzoate
ADCC: Antibody-dependent cell-mediated cytotoxicity
CDC: Complement-dependent cytotoxicity
ROS: Reactive oxygen species
PS: Photosensitizers
NK: Natural killer
CTL: Cytotoxic T lymphocytes
IL15: Interleukin-15
CAR-T: Chimeric antigen receptor T cell
LV: Lentiviral vectors
%IA/g: % Injected activity per gram tissue
%ID/g: % Injected dose of per gram tissue
